# Rhizoxin and 2,4-diacetylphloroglucinol contribute to biocontrol of *Pseudomonas protegens* Pf-5 against pea Ascochyta blight pathogen *Didymella pinodes*

**DOI:** 10.1128/spectrum.01325-26

**Published:** 2026-08-11

**Authors:** Jepri Agung Priyanto, Chiseche Mwanza, Maria Purnamasari, Xiaogang Wu, Li Huang, Qing Yan

**Affiliations:** 1 Department of Plant Sciences and Plant Pathology, Montana State University 33052, Bozeman, Montana, USA; 2 Division of Microbiology, Department of Biology, Faculty of Mathematics and Natural Sciences, IPB University 64759, Bogor, Indonesia; 3 Guangxi Key Laboratory of Agro-Environment and Agro-Product Safety/College of Agriculture, Guangxi University 12664, Nanning, China; Instituto de Ecología, A.C. (INECOL), Michoacán, Mexico

**Keywords:** *Didymella pinodes*, biocontrol, *Paenibacillus*, *Pseudomonas*, antibiotics, DAPG, rhizoxin

## Abstract

Biological control using beneficial bacteria is a promising strategy for managing pea Ascochyta blight (AB), yet the underlying mechanisms remain poorly understood. In this study, we identified 10 bacterial strains from 4 genera, including *Bacillus*, *Paenibacillus*, *Peribacillus*, and *Pseudomonas*, that significantly reduced the severity of AB caused by *Didymella pinodes* under greenhouse conditions. Most strains inhibited *D. pinodes* in cultures, indicating a production of antimicrobial metabolite(s) by these bacteria. To identify the antimicrobial compounds responsible for inhibition of *D. pinodes* and investigate the biocontrol mechanisms, we used *Pseudomonas protegens* Pf-5, which produces eight known antimicrobial compounds, as a model. While wild-type Pf-5 strongly inhibited *D. pinodes* in cultures and controlled AB *in planta*, a derivative (Δ8-fold mutant) lacking all eight compounds showed significantly compromised biocontrol efficacy. Individual complementation of biosynthetic genes for rhizoxin, 2,4-diacetylphloroglucinol (DAPG), pyrrolnitrin, or hydrogen cyanide partially restored inhibitory activity, confirming their roles in inhibition of *D. pinodes*. Notably, restoring rhizoxin and DAPG biosynthesis recovered the disease control capability of the Δ8-fold mutant in greenhouse trials. These results demonstrate that rhizoxin and DAPG are key metabolites driving the biocontrol activity of *P. protegens* Pf-5 against *D. pinodes*.

**IMPORTANCE:**

An advanced understanding of how beneficial bacteria control plant diseases can help us better use these microorganisms in agriculture. In this study, beneficial bacteria were identified to mitigate damages of pea Ascochyta blight (AB) caused by the fungal pathogen *Didymella pinodes*. Most of the identified beneficial bacteria inhibited the fungal pathogen in cultures. Using the soil beneficial bacterium *Pseudomonas protegens* Pf-5 as a model, we demonstrated that four bacteria-derived antimicrobial compounds, rhizoxin and 2,4-diacetylphloroglucinol (DAPG), pyrrolnitrin, and hydrogen cyanide, play important roles in inhibiting *D. pinodes* growth. This study also showed that rhizoxin and DAPG produced by Pf-5 contribute to the suppression of AB disease development. These findings provided new insights into the molecular basis of beneficial bacteria-mediated disease suppression of pea Ascochyta blight.

## INTRODUCTION

Biocontrol using beneficial bacteria is promising in plant disease management. However, application of beneficial bacteria is often hampered by varied efficacy *in planta*. Identification of bacteria that have consistent disease control efficacy is critical, and an advanced understanding of their disease control mechanisms can help us better use the beneficial bacteria to control plant diseases.

Pea Ascochyta blight (AB) is a foliar disease caused by several fungal pathogens including *Didymella pinodes* (synonym: *Ascochyta pinodes*, *Peyronellaea pinodes*), *D. pinodella*, *D. pisi*, and *Ascochyta koolunga* (1–4). These pathogens can infect pea plants individually or coexist as a complex within a single field or plant (5). Among these pathogens, *D. pinodes* has a broad range of hosts including pea (*Pisum sativum*) and at least 19 other legume species (6). *D. pinodes* was reported as the primary pathogen of AB in many pea-growing countries, including Australia (7), Canada (8), China (9), France (10), and the United States (11, 12).

Several bacterial strains have been identified to control pea AB. However, the mechanisms of how beneficial bacteria control pea AB remain largely unknown. For example, *Pantoea agglomerans*, *Bacillus amyloliquifaciens,* and *Bacillus subtilis* significantly reduced pea AB disease severity (9). These bacteria inhibited *D. pinodes* in cultures although the antimicrobial compound(s) were not identified, and their roles in disease control were not determined (9). Additionally, application of *Bacillus megaterium* and *Pseudomonas fluorescens* reduced pea AB disease severity, but the mechanisms remain unclear (13).

Pea AB has been reported in Montana (12), highlighting the need for effective disease management strategies. Indigenous bacteria are often well adapted to local abiotic and biotic conditions (14), enabling them to compete with pathogens and other microorganisms for nutrients and spaces. Previous studies have demonstrated the effectiveness of indigenous bacteria in controlling plant diseases (15–18). Isolating and characterizing beneficial bacteria from pea farms in Montana is expected to yield effective biocontrol agents to control pea AB.

The first goal of this study was to identify beneficial bacteria that control pea AB caused by *D. pinodes*. To achieve this goal, bacterial strains were isolated from pea tissues and soils collected in Montana. Beneficial bacteria that strongly reduced AB disease severity were identified. The second goal of this study was to investigate the molecular mechanisms of biocontrol against *D. pinodes*. To this end, *Pseudomonas protegens* Pf-5, a well-characterized plant beneficial bacterium (19–21), was used as a model strain in this work. Pf-5 produces at least eight antimicrobial compounds, including pyrrolnitrin (22), hydrogen cyanide (23), 2,4-diacetylphloroglucinol (DAPG) (24), pyoluteorin (25, 26), orfamide A (27), rhizoxin (28, 29), toxoflavin (30), and polyyne (31). Production of these compounds is activated by a global regulator GacA in Pf-5 (20, 30, 32, 33). Previous research indicated that Pf-5 could control pea AB although the underlying mechanism was not investigated (34). In this study, the antimicrobial compounds required for Pf-5 to inhibit *D. pinodes* were determined, and their roles in pea AB control were characterized.

## MATERIALS AND METHODS

### Microbial materials and growth conditions

*D. pinodes* isolate C6 was isolated previously from Ascochyta blight (AB)-contaminated pea seeds (34). To promote conidia production, the isolate was routinely cultured on pea meal agar (PMA) at 28°C, prepared by combining 25 g of blended dry pea seeds and 15 g of agar (BD Difco, Becton Dickinson and Company) in 1 L of MiliQ water.

Biocontrol bacteria used in this study include the ones isolated from pea and camelina plants and associated rhizosphere soils in this work and the model strain *P. protegens* Pf-5 (22). Pea-associated bacteria were isolated from rhizosphere soils, leaves, and stems collected from pea farms in Montana. Rhizosphere soils were collected by uprooting plants, removing the attached surface soils via gently shaking the roots, and collecting the firmly attached rhizosphere soils by soaking the roots in water (1 g of the root samples in 10 mL sterilized MiliQ water) with vortexing for around 2 min. The leaf- and stem-associated bacteria were isolated by homogenizing 1 g of the plant tissues in 10 mL sterilized MiliQ water. A 100-µL aliquot of the suspension was spread onto various culture media, including Reasoner’s 2A agar (R2A, Neogen), potato dextrose agar (PDA, BD Difco, Becton Dickinson and Company), and tryptic soy agar (TSA, BD Difco, Becton Dickinson and Company). The culture plates were incubated at 28°C for 48 h before the isolates were picked. A total of 250 isolates were collected from the pea plants. Additionally, 100 isolates were collected from camelina rhizosphere soils using a similar method. These 350 isolates were stored in Luria Bertani (LB) medium (composition g/L: 10 g of tryptone, 5 g of yeast extract, 10 g of NaCl, 1 L of deionized water) supplemented with 20% glycerol at −80°C for further study.

*P. protegens* Pf-5 was originally isolated from cotton field soils (22). Pf-5 and its derivatives used in this study were listed in Table 1. The bacterium was cultured at 28°C in King’s B medium (KB) (35) unless other specific media were described.

**TABLE 1:** Bacterial strains and primers

Strains	Genotypes, relevant characteristics^*a*^, DNA sequences	References
LK099	Wild-type strain P. protegens Pf-5, produces multiple antibiotics. DAPG^+^, Ofa^+^, Prn^+^, Plt^+^, HCN^+^, Rzx^+^, Pgn^+^, Tox^+^	(21)
JL4975	Pf-5 derivative strain contains an in-frame deletion *gacA* of in the chromosome, changed in many of the phenotypes controlled by GacA. DAPG^−^, Ofa^−^, Prn^−^, Plt^−^, HCN^−^, Rzx^−^, Pgn^−^, Tox^−^	(36)
LK891	Δ7-fold mutant, Pf-5 derivative strain. Δ*prnC*, Δ*rzxB*, Δ*pltA*, Δ*hcnB*, Δ*ofaA*, Δ*phlA*, Δ*pgnE*. Prn^−^, Rzx^−^, Plt^−^, HCN^−^, Ofa^−^, DAPG^−^, Pgn^−^	This study
LK892	Δ8-fold mutant, Pf-5 derivative strain. Δ*prnC*, Δ*rzxB*, Δ*pltA*, Δ*hcnB*, Δ*ofaA*, Δ*phlA*, Δ*pgnE*. Δ*toxD*, Prn^−^, Rzx^−^, Plt^−^, HCN^−^, Ofa^−^, DAPG^−^, Pgn^−^, Tox^−^	This study
LK929	Δ8-fold/*rzxB*^+^ LK892 containing a restored *rzxB* gene in the chromosome, made by introducing a plasmid pEX18Tc-*rzxB* (ID L6472) into LK892. This derivative strain produces rhizoxin	This study
LK883	Δ8-fold/*phlA*^+^ LK892 containing a restored *phlA* gene, made by introducing a plasmid pME6010-*phlA* (ID LK880) into LK892. This derivative strain produces DAPG	This study
LK884	Δ8-fold/*hcnB*^+^ LK892 containing a restored *hcnB* gene, made by introducing a plasmid pME6010-*hcnB* (ID LK881) into LK892. This derivative strain produces hydrogen cyanide	This study
LK930	Δ8-fold/*toxD*^+^ LK892 containing a restored *toxD* gene, made by introducing a plasmid pME6010-*toxD* (ID LK882) into LK892. This derivative strain produces toxoflavin	This study
LK931	Δ8-fold/*pgnE*^+^ LK892 containing a restored *pgnE* gene, made by introducing a plasmid pME6010-*pgnE* (ID LK821) into LK892. This derivative strain produces polyyne	This study
LK933	Δ8-fold/*pltA*^+^ LK892 containing a restored *pltA* gene, made by introducing a plasmid pME6010-*pltA* (ID LK932) into LK892. This derivative strain produces pyoluteorin	This study
LK934	Δ8-fold/*ofaA*^+^ LK892 containing a restored *ofaA* gene, made by introducing a plasmid pME6010-*ofaA* (ID LK879) into LK892. This derivative strain produces orfamide	This study
LK935	Δ8-fold/*prnC*^+^ LK892 containing a restored *prnC* gene, made by introducing a plasmid pME6010-*prnC* (ID LK878) into LK892. This derivative strain produces pyrrolnitrin	This study
LK937	Δ8-fold/*phlA*^+^ LK892 containing a restored *phlA* gene in the chromosome, made by introducing a plasmid pEX18Tc-*phlA* (ID L6471) into LK892. This derivative strain produces DAPG	This study
LK938	Δ8-fold/ *rzxB*^+^ *phlA*^+^ LK892 containing restored *rzxB* and *phlA* gene in the chromosome, made by introducing a plasmid pEX18Tc-*phlA* (ID L6471) into LK929. This derivative strain produces rhizoxin and DAPG	This study

Primers	Oligonucleotide sequences (5′−3′)^*b*^	References
P19	AGAGTTTGATCCTGGCTCAG	(37)
P20	CGGTTACCTTGTTACGACTT	(37)
P646	GCDGCHGCNATGCGTTAYAC	(38)
P647	ACAAGMTCWGCKATTTTTTC	(38)
P644	TGGCCGAGAACCAGTTCCGCGT	(39)
P645	CGGCTTCGTCCAGCTTGTTCAG	(39)
P264	ATAGGTACCTGATCATCATTGCCGGG	(33)
P265	TATAAGCTTGAACGCACGATGGGCCAG	(33)
P532	ATAGGTACCGTCAACTCCTTCGGCTTGTC	This study
P533	ATAGGTACCCTATTTGGAGTCTGCTGT	This study
P534	TATAAGCTTTTCACATTCAGTGCTGGAGC	This study
P535	ATAGGTACCTCCACGTACTCAGCGAAGAA	This study
P536	TATAAGCTTCGCTGGGAGTTGAAGACATC	This study
P537	ATAGAATTCGATGAACCCACAATGCGTCA	This study
P538	TATAAGCTTCGGTCTGCTCGAACTTGAAA	This study
P539	TATAAGATCTCCGGAGCTGGAAATCAACTG	This study
P542	TATAAGCTTAGCATGTCCGGGGTGATC	This study
P543	TATAAGATCTATGTTGTCGGGGCTGTTTTG	This study
P544	ATAGAATTCGACAAAATCGGTCCCAGCAC	This study

^*a*^ DAPG, 2,4-diacetylphloroglucinol; Ofa, orfamide; Prn, pyrrolnitrin; Plt, pyoluteorin; HCN, hydrogen cyanide; Rzx, rhizoxin; Pgn, polyyne; Tox, toxoflavin.

^*b*^ Underlined nucleotides in primer sequences represent restriction enzyme recognition sites introduced for cloning purposes.

### Plant materials and growth conditions

The seeds of pea (*Pisum sativum*) variety Fallon were obtained from the Montana State University (MSU) Pulse Breeding Program. The seeds were surface-sterilized with 3% sodium hypochlorite for 2 min, rinsed three times with sterile water, and air-dried prior to sowing in Sunshine soil, a peat-based potting mix (Sun Gro Horticulture, Agawam, MA). The soil was placed in a pot (9 cm diameter × 9 cm height). Plants were then grown in the greenhouse at the MSU Plant Growth Centre under controlled conditions, including a day/night temperature of 30.2/18°C, 22% relative humidity, and a 16-h photoperiod. After 2 weeks of growth, the plants were considered ready for inoculation.

### Biocontrol assays

#### Preparation of inocula and plant inoculation procedures

Bacteria strains were cultured overnight at 28°C on LB (BD Difco, Becton Dickinson and Company) plates or KB plates. Cells were harvested from the plates and washed three times with sterile water. Approximately 20 mL of each bacterial suspension (OD_600_ = 1.0), amended with one drop of Tween 20, was spray-inoculated onto upper and lower leaf surfaces of the 2-week-old pea plants until the leaf surfaces were uniformly coated and visibly wet. The plants were maintained in a dew chamber at 22°C for 24 h prior to *D. pinodes* inoculation.

A mixed inoculum of the beneficial bacteria was prepared by mixing *P. protegens* Pf-5 and the individual pea-associated bacterial isolate, yielding 20 mL of a mixed bacterial suspension (final cell density at OD_600_ = 1.0 for both strains). At this optical density, the cell concentration was quantified to be approximately 1.5 × 10^9^–6 × 10^9^ CFU/mL for *Pseudomonas* spp., and 3.5 × 10^7^–3.9 × 10^8^ CFU/mL for *Bacillus*, *Peribacillus*, and *Paenibacillus* spp., as determined using spread plate procedure. The mixed inoculum was spray-inoculated onto both the upper and lower leaf surfaces as described above.

To prepare the pathogen inoculum, *D. pinodes* was cultured on PMA plates for 2 weeks at 28°C. The culture plate was flooded with 30 mL of sterile distilled water and gently scraped with a glass rod to dislodge conidia. The conidia suspension was filtered through a double layer of sterilized cheesecloth. The number of conidia was counted using hemocytometer (Blau Brand, Sigma-Aldrich, Germany) under a compound microscope (DM750, Leica Microsystems, Germany), adjusted to 1 × 10^5^ conidia/mL in 200 mL of water supplemented with two drops of Tween 20, and then spray-inoculated onto the pea leaves. The pea plants were placed in a dew chamber at 22°C in darkness for 24 h before being transferred to a greenhouse and incubated in a plastic tent with day/night temperature of 27/18°C, 83.6% humidity, and 16 h of light. Plants sprayed only with sterile water or the pathogen served as negative and positive controls, respectively. Each treatment consisted of 10 pots with one seed per pot. The experiment was repeated independently three times.

#### Disease evaluation

**Fig 1:**
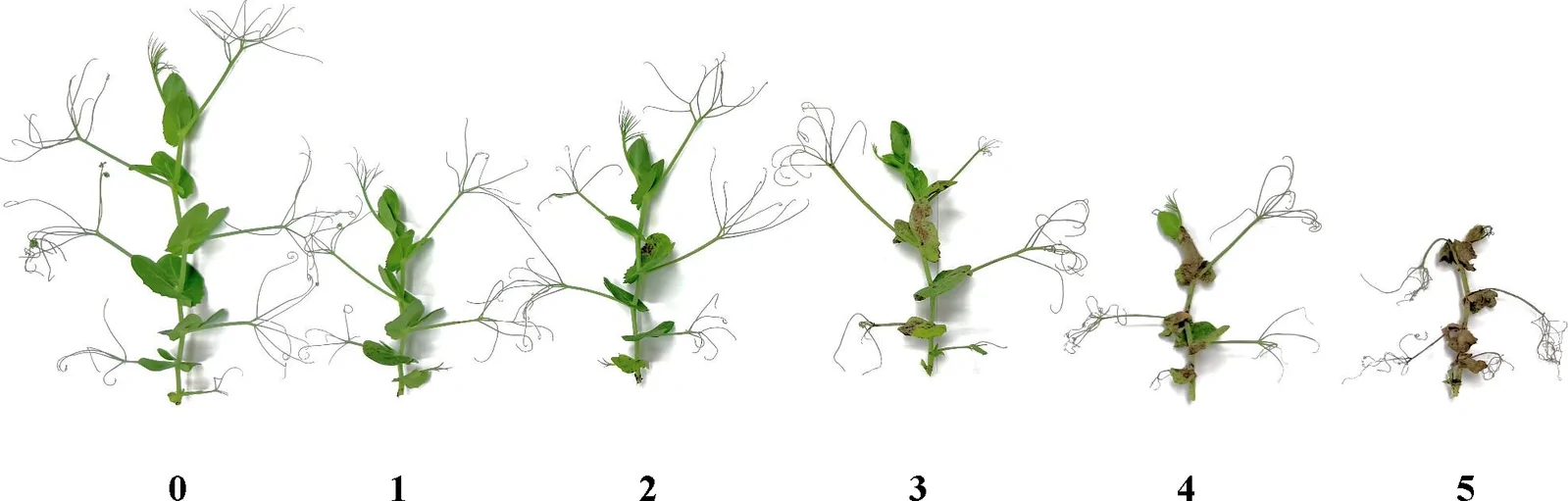
Photos of pea plants representing each level (0–5) of the standard disease severity index (DSI).

The AB disease was evaluated at 7 days after pathogen inoculation. The severity of symptoms on leaves, stems, and stipules was assessed using a Disease Severity Index (DSI) including a scale of the disease severity between 0 and 5 as shown in Fig. 1 and described below:

0 = No visible lesions on any part of the plant; plant appears healthy.

1 = A few small leaf flecks.

2 = Less than 25% of the plant leaves show lesions; the stem is unaffected.

3 = Numerous flecks or lesions on leaves and/or stems; less than 50% coverage.

4 = Large, coalescing lesions covering more than 50% of the plant surfaces; plant still alive.

5 = Severe lesions with stem girdling; plant is dead.

#### Assessment of bacterial persistence and duration of biocontrol activity on leaves

Pea leaves were spray-inoculated with bacterial suspensions (OD_600_ = 1, equivalent to 1.5 × 10^9^ CFU/mL for *P. protegens* Pf-5, and 2.7 × 10^8^ CFU/mL for LK829) until evenly wetted and then incubated for 24 h in a dew chamber. Subsequently, *D. pinodes* spores were applied at three time points post-bacterial application: 1, 7, or 14 days. DSI was measured 7 days after pathogen inoculation. The bacterial populations on leaves were also quantified: 1 g of inoculated leaves was mixed with 1 mL of sterile water, three glass beads, and homogenized at 400 rpm for 1 min using bead-beating microtube homogenizer (BeadBugTM 3, Benchmark Scientific, D1030, USA). The bacterial populations were quantified by spreading a serial dilutions of the bacterial suspension on KB plates supplemented with 100 µg/mL streptomycin for Pf-5 that has a natural resistance to streptomycin (40), and on LB plates supplemented with 100 µg/mL ampicillin for LK829. Bacterial populations were expressed as CFU/g of leaf tissues.

### Inhibition assays of selected isolates against *D. pinodes*

The inhibition of beneficial bacteria against *D. pinodes* was evaluated using two different methods: mycelium-based inhibition assay and spore-based inhibition assay, as described below.

#### Mycelium-based inhibition assay

A 6-mm mycelial plug from a 7-day-old *D. pinodes* culture was placed at the center of a PMA plate and incubated at 28°C for 7 days. Subsequently, 3 µL of bacterial suspension (OD_600_ = 1, equivalent to around 10^9^ CFU/mL for *Pseudomonas* spp., around 10^8^ CFU/mL for *Peribacillus* sp. and *Paenibacillus* spp., and around 10^7^ CFU/mL for *Bacillus* spp.) was spotted 2.5 cm from the edge of the fungal colony. The plates were allowed to air-dry and then incubated for an additional 7 days at 28°C before the inhibition zone was measured.

#### Spore-based inhibition assay

Conidia from a 2-week-old *D. pinodes* culture were harvested by flooding the culture with sterile water and filtering the suspension through six layers of sterile Miracloth (Millipore Sigma). A 200-µL aliquot of the conidia suspension (1 × 10^5^ conidia/mL) was added to 20 mL of molten PDA (50°C), resulting in a final concentration of 1 × 10^3^ conidia/mL. The mixture was then gently poured into Petri dishes. Once the medium solidified, 3 µL of bacterial suspension (OD_600_ = 3, equivalent to around 3 × 10^9^ CFU/mL for *Pseudomonas* spp, 3 × 10^8^ CFU/mL *Paenibacillus* spp., and *Peribacillus* sp., and around 3 × 10^7^ CFU/mL for *Bacillus* spp.) was spotted onto the agar surface and incubated at 28°C for 3 days. Using the same procedure, inhibitory activity was also evaluated on pea leaf agar (PLA). PLA was prepared by homogenizing 10 g of fresh pea leaves in 100 mL of distilled water, followed by filtration through two layers of Miracloth (MilliporeSigma) to obtain a leaf extract. A total of 100 mL of this filtrate was then mixed with 100 mL of distilled water containing 1 g of blended dry pea seeds. The medium was subsequently autoclaved prior to use.

The results of both assays were evaluated by measuring the inhibition zone between the edge of the bacterial colony and the fungal colony. The assays were performed in three independent experiments, each with two replicates.

### Taxonomic characterization of beneficial bacterial isolates

Bacterial genomic DNA was extracted using Monarch Spin gDNA Extraction kit (New England Biolabs). All bacterial isolates were initially identified based on their 16S rRNA gene sequences by following a previous study (37). Isolates of *Pseudomonas* spp. and *Bacillus* spp. were further analyzed by sequencing *rpoB* and *gyrA*, respectively. Each gene was amplified by PCR using primer pairs 16Sr RNA (P19/P20), *rpoB* (P644/p645), *gyrA* (P646/P647) as developed by previous studies (37–39) (Table 1). PCR amplification was carried out in a 50-µL reaction mixture containing 2 µL of genomic DNA (100 ng/µL), 2 µL of each primer, 25 µL of KOD 2 × Master Mix, and 19 µL of deionized water. PCR conditions were as follows: initial denaturation at 95°C for 5 min; followed by 35 cycles of denaturation at 95°C for 30 s, annealing at 55°C (16S rRNA), 50°C (*gyrA*), or 54°C (*rpoB*) for 30 s, and extension at 72°C for 40 s; with a final extension at 72°C for 5 min. The expected amplicon sizes for 16S rRNA, *gyrA,* and *rpoB* are 1,492, 500, and 1,247 bp, respectively. All the sequences were deposited to NCBI GenBank and can be accessed via the accession numbers listed in Fig. 1E. Multigene alignment was performed using the MUSCLE algorithm, and the phylogenetic tree was constructed using the maximum likelihood method in Molecular Evolutionary Genetics Analysis (MEGA) version 12.1.

### Construction of *P. protegens* Pf-5 derivatives

#### Construction of Pf-5 mutants

The Δ7-fold mutant (Δ*pltA*Δ*prnC*Δ*ofaA*Δ*rzxB*Δ*hcnB*Δ*phlA*Δ*toxD*, strain LK891) was made by deleting *toxD***,** using a deletion construct pEX18Tc-Δ*toxD* (30), from the chromosome of a Δ6-fold mutant (Δ*pltA*Δ*prnC*Δ*ofaA*Δ*rzxB*Δ*hcnB*Δ*phlA*, strain LK147) (41). Next, the Δ8-fold mutant (Δ*pltA*Δ*prnC*Δ*ofaA*Δ*rzxB*Δ*hcnB*Δ*phlA*Δ*toxD*Δ*pgnE*, strain LK892) was made by deleting *pgnE***,** using a deletion construct pEX18Tc-Δ*pgnE* (33), from the chromosome of LK891. The mutants were generated following our previous protocol (42). Briefly, the deletion constructs were introduced into Pf-5 cells via electroporation (1.8 kV, 5 ms). Single-crossover recombinants were selected on KB amended with tetracycline, followed by selection of double-crossover recombinants on LB amended with sucrose to resolve the plasmid backbone using the SacB-based counterselection. The deletions of target genes were confirmed by whole sequencing analysis as described below.

#### Whole-genome sequencing of Pf-5 mutant to validate mutations

The genome of the Δ8-fold mutant (strain LK892) was sequenced to confirm the mutation of target genes for the eight antimicrobial compounds. Genomic DNA was extracted using the MasterPure Complete DNA and RNA Purification Kit (Lucigen, Middleton, WI). Long-read whole-genome sequencing was performed on an Oxford Nanopore MinION platform using a standard ligation-based library preparation workflow and ONT flow cell chemistry. Raw reads were basecalled with ONT basecalling software, and adapter/low-quality sequences were removed prior to assembly. A total of 532,268 raw reads were generated and used for genome assembly on the Tempest high-performance computing cluster using a standard long-read assembly pipeline, followed by polishing to improve consensus accuracy. The final assembly was annotated with Bakta, and circular genome maps (CDS, RNA features, GC content, and GC skew) were generated using Proksee (43).

To confirm the mutation of the target eight genes, sequences spanning each candidate antibiotic biosynthetic gene were extracted from the assembled mutant genome and compared to the genome of the wild-type Pf-5 using NCBI BLAST searches. BLAST alignments were used to identify the corresponding Pf-5 orthologs and to define the mutations in the Δ8-fold mutant (strain LK892). Coordinates from the Pf-5 reference were used to report mutation boundaries and to generate locus schematics for the eight antibiotic-associated genes. To confirm that the Δ8-fold mutant genotype was otherwise conserved, we performed a genome-wide comparison against the Pf-5 wild-type reference and screened the variant list for additional high-confidence disruptive changes; no other mutations with clear predicted loss-of-function effects were detected beyond the eight antibiotic-associated loci.

#### Construction of complemented strains of Pf-5

Individual complementation of seven antibiotic biosynthetic genes (*phlA*, *prnC*, *toxD*, *hcnB*, *ofaA*, *pltA*, *pgnE*) was made by introducing the corresponding wild-type genes, carried by pME6010 vector, into the Δ8-fold mutant. Each of the genes was amplified by PCR using the following primer pairs: *phlA* (P533/P534), *prnC* (P531/P532), *toxD* (P535/P536), *hcnB* (P537/P538), *ofaA* (P539/P542), *pltA* (P543/P544), and *pgnE* (P264/P265). The constructs were verified by sequencing and introduced into LK892 by electroporation (1.8 kv, 5 ms). The double complementation of *phlA* and *rzxB* in the Δ8-fold mutant was made through chromosomal replacement using pEX18Tc-based constructs containing the wild-type *phlA* (44) or *rzxB* (36). The chromosomal replacement was performed following the above method of gene deletion. The complementation was confirmed by PCR analysis and phenotype assays as described below.

Restored production of the eight antimicrobial compounds in the Δ8-fold mutant was further validated by phenotypic assays, including direct visualization of the compounds via high-performance liquid chromatography (HPLC) analysis, inhibition against microorganisms sensitive to the compounds, and other bacterial traits that rely on the compounds. Specifically, Δ8-fold/*rzxB*^+^ was tested via inhibition of *D. pinodes* which is sensitive to rhizoxin (this study); Δ8-fold/*phlA*^+^ was tested via inhibition of *Aphanomyces euteiches* which is sensitive to DAPG (20); Δ8-fold/*toxD*^+^ and Δ8-fold/*pgnE*^+^ were tested via inhibition of *Pseudomonas syringae* which is sensitive to toxoflavin and polyyne (30, 33). Specifically, Δ8-fold/*toxD*^+^ was cultured on KB plus 0.1 mM FeCl_3_ plates to test its production of toxoflavin (30); Δ8-fold/*pgnE*^+^ was cultured on NA plus 1% glycerol plates to test its production of polyyne (33). Δ8-fold/*ofaA*^+^ was tested via a swarming motility assay on 0.6% agar M9 minimal medium because orfamide is needed in bacterial motility (45); Δ8-fold/*hcnB*^+^ was tested via a picric acid-based colorimetric assay (46). Strains Δ8-fold/*phlA*^+^, Δ8-fold/*pltA*^+^ and Δ8-fold/*prnC*^+^ were cultured in potato dextrose broth (PDB) (BD Difco, Becton Dickinson and Company) for 24 h, in nutrient yeast broth supplemented with 0.35 mM zinc sulfate and 1% of glycerol (NYBGly-Zn) for 48 h (47), and on pea meal agar for 7 days, respectively. DAPG was extracted from supernatant using ethyl acetate. Pyoluteorin was extracted from supernatant after acidification to pH 2.0 with HCl and subsequently dissolved in ethyl acetate. Pyrrolnitrin was extracted from bacterial cells grown on solid culture as previously described (23, 40), with slight modifications. The cells were suspended in 80% acetone, sonicated on ice, and subsequently extracted with ethyl acetate. All ethyl acetate phases were then dried in a vacuum evaporator (Vacufuge, Eppendorf, Germany) at 45°C. Target compounds were detected using HPLC under conditions described previously (32). The presence of each compound was confirmed by comparison with the authentic standards, including DAPG (Thermo Fisher Scientific, USA), pyrrolnitrin (Sigma-Aldrich, Israel), and purified pyoluteorin (48).

### Statistical analysis

Statistical analysis was performed using R software (version 4.5.3). One-way ANOVA was used for single-factor experiments, followed by post hoc comparisons at *P* < 0.05. Disease severity index (DSI) was analyzed using the Kruskal-Wallis test, whereas bacterial populations, inhibition zone radii, and fungal colony diameters were analyzed using Tukey’s HSD test.

## RESULTS

### Identification of bacterial isolates with biocontrol activity

**Fig 2:**
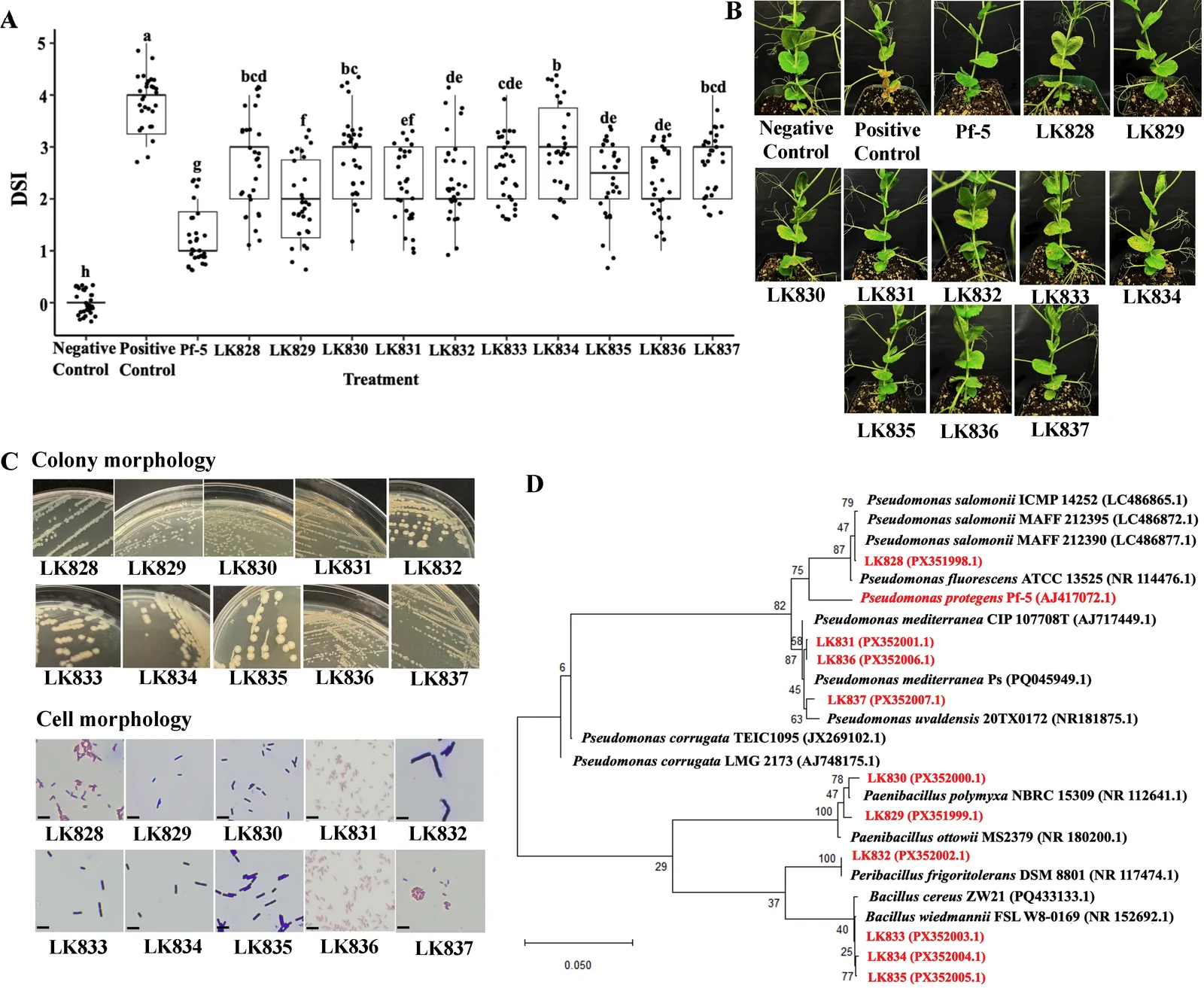
Biocontrol activity of beneficial bacteria against pea Ascochyta blight. (**A**) Disease severity index (DSI) of pea plants treated with *P. protegens* Pf-5 and the other bacterial isolates. Disease was evaluated 7 days after inoculation of *D. pinodes*. Data were presented as mean ± standard deviation. Each dot represents an individual plant. A total of 30 plants were used in each treatment. The experiment was repeated three times independently. Different letters indicate significant differences among treatments (ANOVA, Kruskal-Wallis test, *P* < 0.05). (**B**) Results of the biocontrol assay. Photos of the representative plant were taken at 7 days after fungal inoculation. (**C**) Colony and cell morphology of the bacterial isolates grown on LB medium at 28°C for 24 h. A cell with a pink or purple color indicates the isolate was identified to be Gram-negative or Gram-positive, respectively, via the Gram staining assay. The bar size is 10 µm. (**D**) Phylogenetic tree of the tested bacterial strains (highlighted in red) constructed via multiple genes (16S rRNA, *rpoB*, *gyrA*) sequences analysis. Numbers at the node indicate the percentage of bootstrap support based on a maximum likelihood method of 1,000 resampled data sets. The scale bar represents 0.05 substitution per nucleotide position.

A total of 350 bacterial strains were isolated from pea and camelina plants grown in Montana and screened for beneficial bacteria to control pea AB caused by *D. pinodes* in greenhouse. The 10 most effective isolates (LK828 –LK837), that showed significant and consistent biocontrol activity in at least three independent experiments, were selected for further investigation (Fig. 2A and B). Gram staining and microscopic analyses revealed that four isolates were Gram-negative and six were Gram-positive (Fig. 2C). Phylogenetic analysis based on sequence alignment of three housekeeping genes (16S rRNA, *rpoB*, and *gyrA*) showed that four isolates (LK828, LK831, LK836, and LK837) belonged to *Pseudomonas*, three (LK833, LK834, and LK835) to *Bacillus*, two (LK829 and LK830) to *Paenibacillus*, one (LK832) to *Peribacillus* (Fig. 2D).

This study also includes a soil bacterium *P. protegens* Pf-5 because of its previously reported biocontrol activity against pea AB (34). Our result shows that Pf-5 exhibited a stronger biocontrol activity than the 10 pea bacterial isolates (Fig. 2A).

### Beneficial bacteria protected pea plant from *D. pinodes* for up to 2 weeks

**Fig 3:**
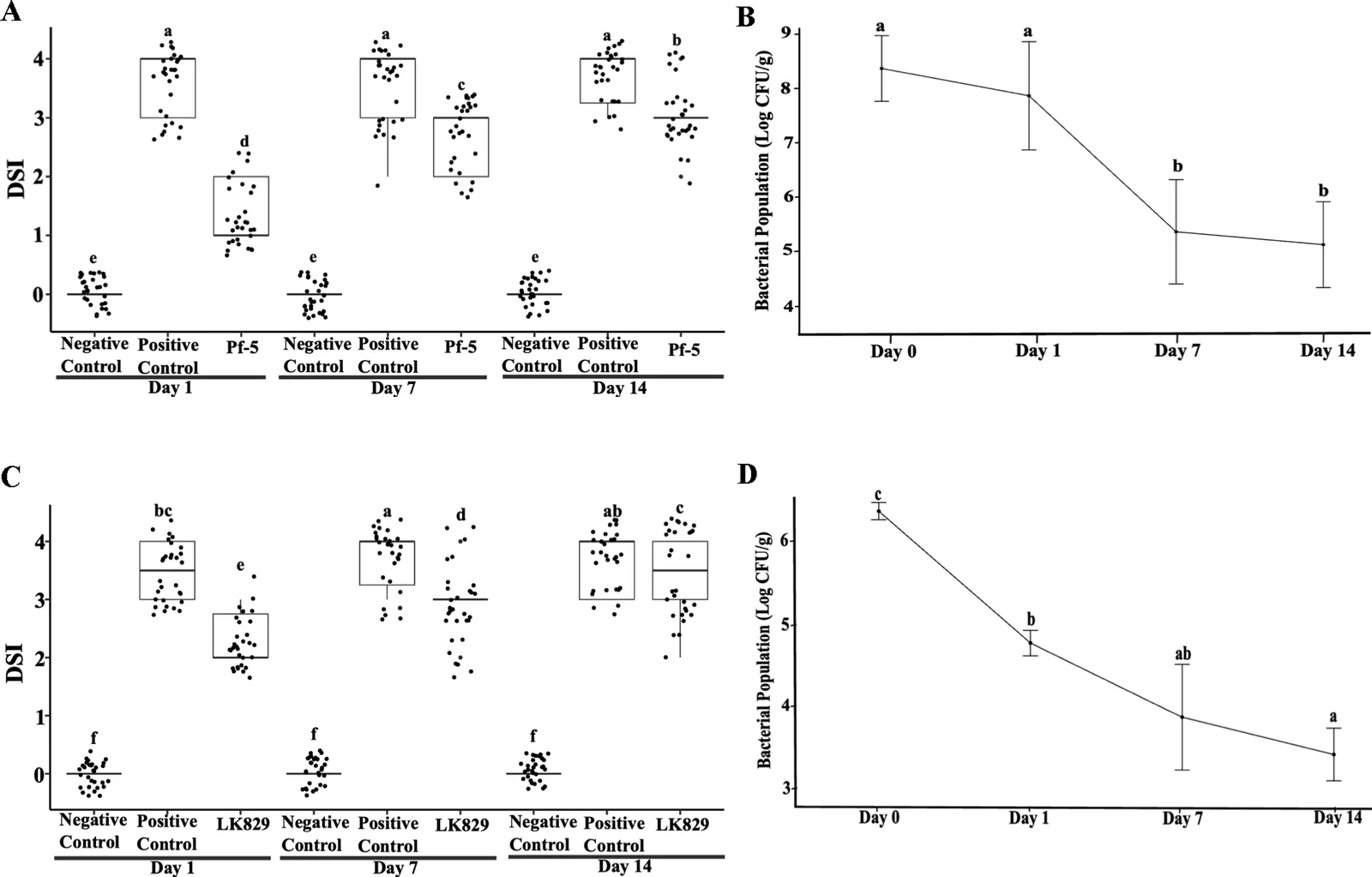
Biocontrol activity against *D. pinodes* at 1, 7, or 14 days after application of *P. protegens* Pf-5 (**A, B**) or *Paenibacillus* sp. LK829 (**C, D**). Each treatment had 10 replicates. The experiment was independently repeated three times. Disease severity index (DSI) of pea plants was evaluated 7 days post fungal inoculation (**A, C**). Leaves were sampled at 0, 1, 7, or 14 days after bacterial application to measure the population sizes of the inoculated bacteria (**B, D**). Data were presented as means ± standard deviation. Different letters indicate significant differences among treatments (ANOVA, Kruskal-Wallis test, *P* < 0.05).

*P. protegens* Pf-5 and *Bacillus* sp. LK829, the most effective Gram-negative and Gram-positive bacterial isolates identified in this study, were further characterized for their biocontrol traits. To evaluate the duration of protection against *D. pinodes*, pea plants treated with the beneficial bacteria were kept in a greenhouse for 1, 7, or 14 days prior to pathogen inoculation. A single application of Pf-5 protected pea plants for up to 14 days (Fig. 3A) although disease suppression declined over time. Pf-5 exhibited the highest biocontrol efficacy immediately after application on pea leaves, with a noticeable decrease in efficacy at the later time points (7 and 14 days). To assess the persistence of Pf-5 on plant, its population density on pea leaves was quantified at multiple time points. The population declined from an initial density of 2.5 × 10^8^ CFU/g to 1.3 × 10^5^ CFU/g by day 14 (Fig. 3B).

A similar disease control profile was observed for strain LK829, which protected pea plants from *D. pinodes* infection for up to 7 days following plant application (Fig. 3C). The population density of LK829 on pea leaves gradually declined from an initial level of 2.5 × 10^6^ CFU/g to 2.5 × 10^3^ CFU/g by day 14 (Fig. 3D).

### Mixed inoculation of different beneficial bacteria did not enhance biocontrol efficacy

**Fig 4:**
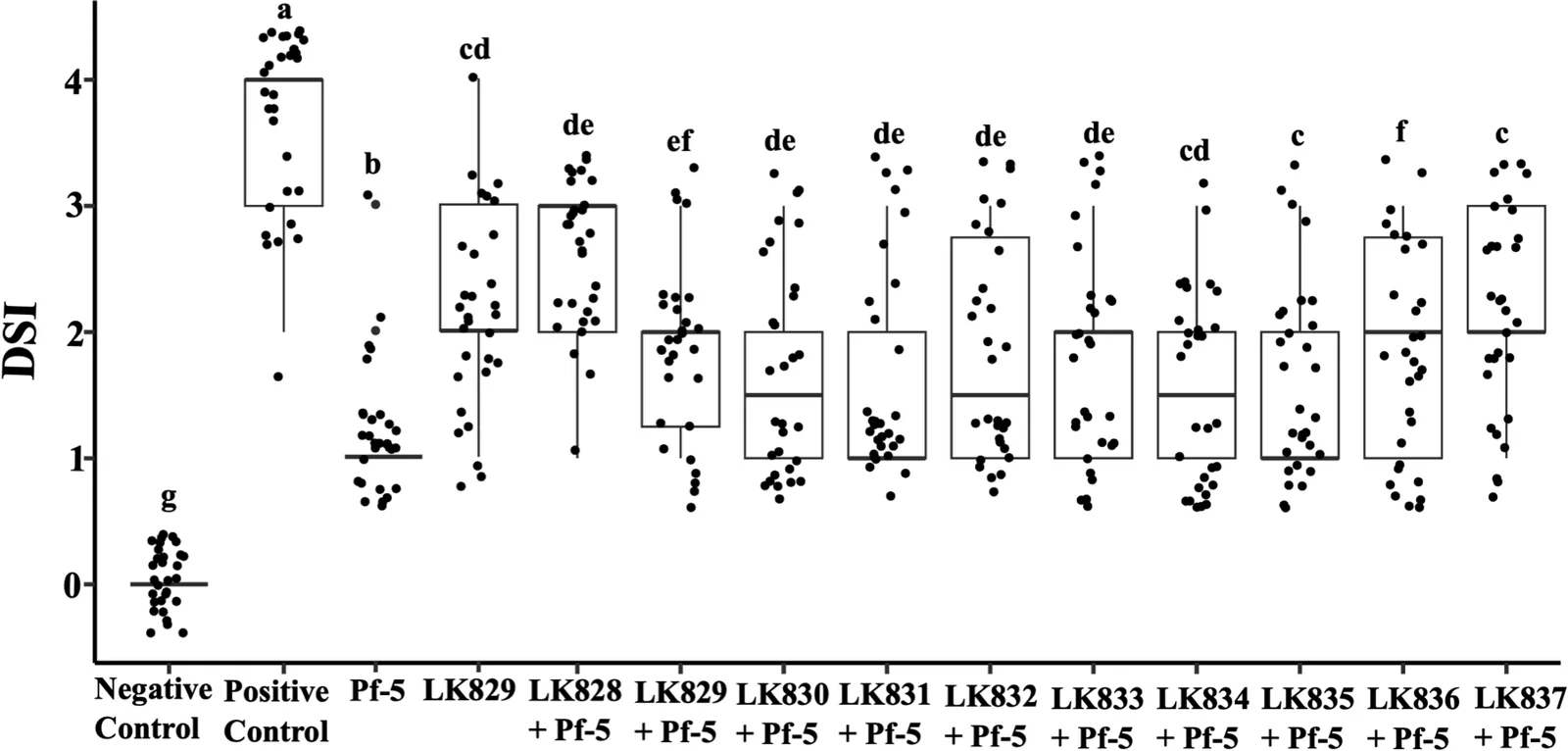
Biocontrol activity of mixed inoculation of the beneficial bacteria. Single application of Pf-5 and LK829 was also included in the assay. The disease severity index (DSI) of each treatment was evaluated at 7 days post fungal inoculation. A total of 30 plants were used in each treatment. Data were presented as means ± standard deviation. Different letters indicate significant differences among treatments (ANOVA, Kruskal-Wallis test, *P* < 0.05). The experiment was repeated three times independently.

To determine whether different beneficial strains could interact synergistically to enhance disease control, Pf-5 was individually mixed with each of the selected pea-associated beneficial bacteria and sprayed onto pea leaves prior to inoculation with *D. pinodes*. Overall, co-inoculation of Pf-5 with any of the 10 pea-associated strains did not improve biocontrol efficacy compared with Pf-5 applied alone (Fig. 4). In fact, co-inoculation with four strains (LK828, LK829, LK836, and LK837) reduced the biocontrol activity of Pf-5, whereas mixtures with the remaining six strains resulted in disease control levels comparable to Pf-5 single inoculation.

A single inoculation of LK829 was included as a reference to facilitate comparison with its mixed inoculation with Pf-5. The combined application of LK829 and Pf-5 provided greater disease suppression than LK829 alone but was less effective than Pf-5 applied individually. Despite these differences, all mixed inoculations resulted in significantly reduced disease symptoms compared with the pathogen-only control.

### Most of the beneficial bacterial isolates inhibited *D. pinodes* in cultures

**Fig 5:**
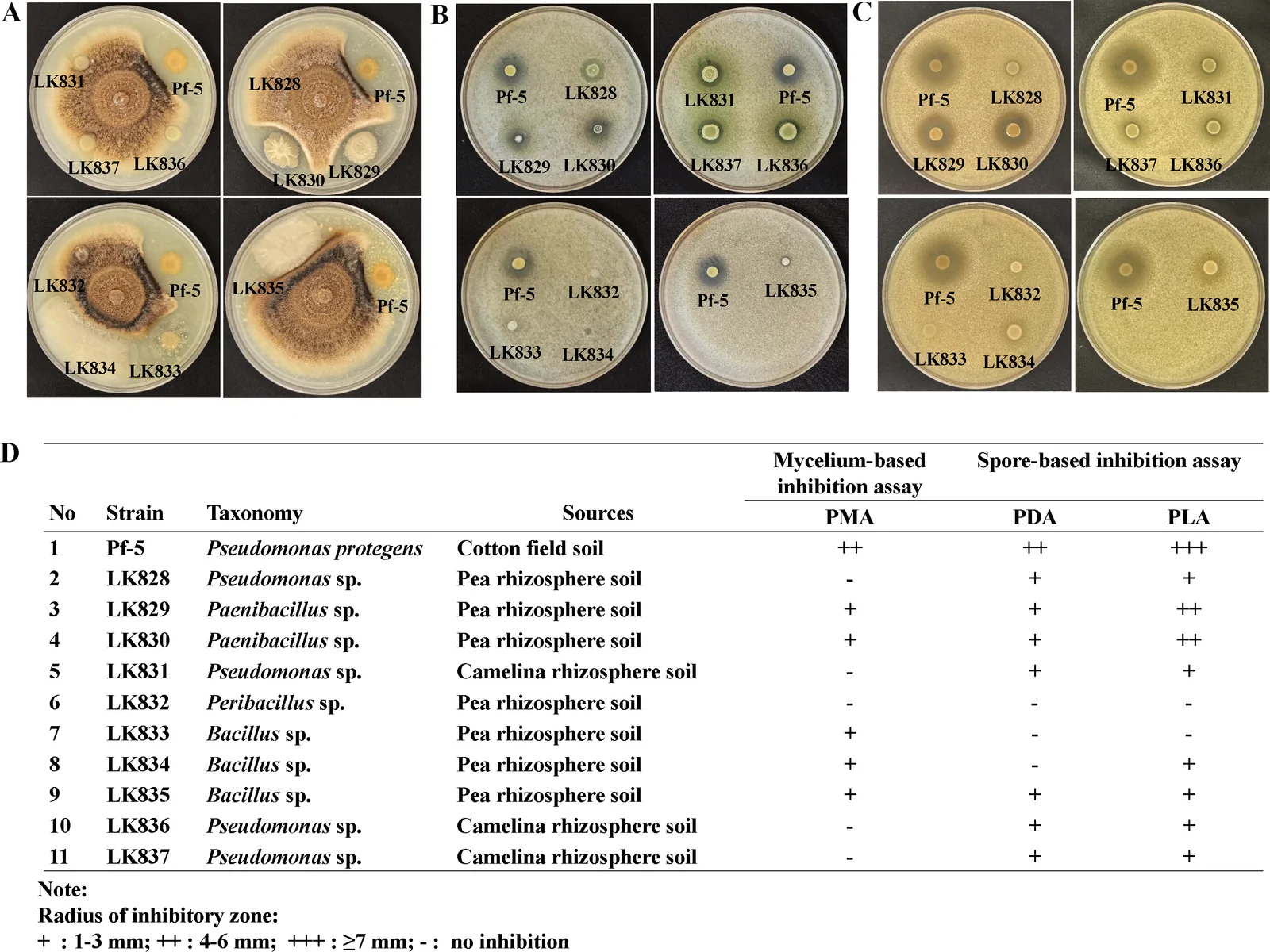
Inhibitory activity of beneficial bacteria against *D. pinodes* in cultures. (**A**) Mycelial growth inhibition assessed on pea meal agar (PMA) 1 week after bacterial inoculation. (**B**) Fungal growth inhibition assessed using a spore-based inhibition assay on PDA plates. (**C**) Fungal growth inhibition on pea leaf agar (PLA). (**D**) Inhibitory activity was scored semi-quantitatively as –, +, ++, and +++, representing no, weak, moderate, and strong inhibition based on the radius of the inhibition zone between the edges of the bacterial and fungal colonies. Images are representative of two replicates. All experiments were independently repeated three times.

To investigate the mechanism used by the beneficial bacteria to control *D. pinodes*, their antagonistic activity against *D. pinodes* was evaluated using both mycelium-based inhibition assay and spore-based inhibition assay. In the mycelium-based inhibition assay, six isolates (Pf-5, LK829, LK830, LK833, LK834, LK835) exhibited inhibitory activity against *D. pinodes* on PMA plates (Fig. 5A). Antagonistic activity was further evaluated using a spore-based inhibition assay, showing that eight isolates inhibited *D. pinodes* on PDA plates (Fig. 5B). To better mimic the nutrient environment of pea leaves, pea leaf agar (PLA) was developed and used in the spore-based inhibition assay. Nine isolates exhibited inhibitory activity on PLA plates (Fig. 5C).

Overall, 10 of the 11 tested bacterial strains, except *Paenibacillus* sp. LK832, inhibited *D. pinodes* under at least one culture condition (Fig. 5D). Notably, four isolates (Pf-5, LK835, LK829, and LK830) displayed inhibitory activity in all assays. One isolate, LK834, showed inhibitory activity only on pea-based media (PMA and PLA).

### Rhizoxin, DAPG, pyrrolnitrin, and hydrogen cyanide contribute to the inhibition of *D. pinodes* by *P. protegens* Pf-5

**Fig 6:**
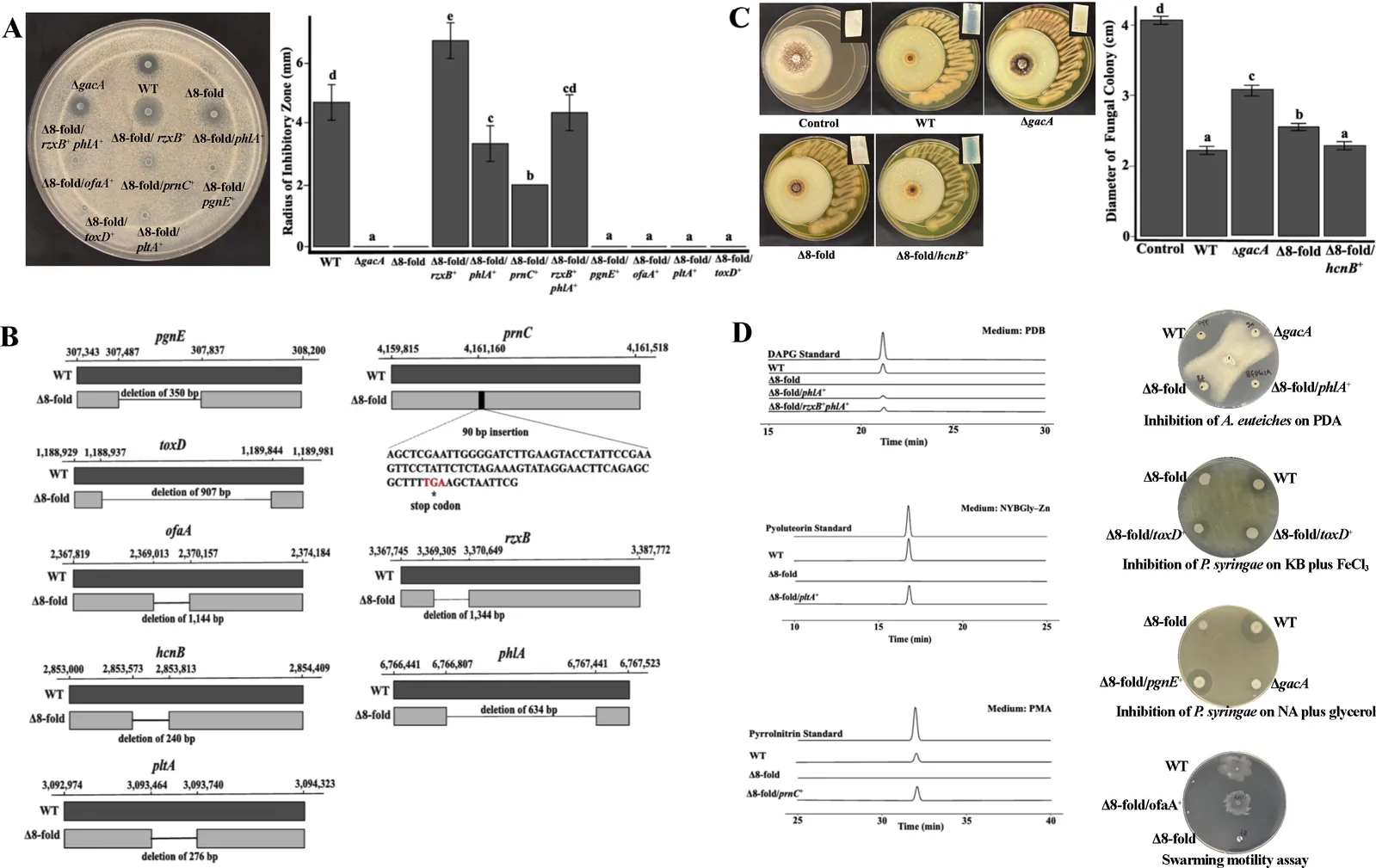
Identification of antimicrobial compounds required for *P. protegens* Pf-5 to inhibit *D. pinodes* in cultures. (**A**) Inhibition of *P. protegens* Pf-5 and its derivatives against *D. pinodes* on a PDA plate. Each bacterial suspension was spot-inoculated onto PDA medium premixed with *D. pinodes* spores. The plate was incubated at 28°C for 3 days before recording the results. WT: wild type; Δ8-fold: Δ*prnC*Δ*rzxB*Δ*pltA*Δ*hcnB*Δ*ofaA*Δ*phlA*Δ*pgnE*Δ*toxC*; Δ8-fold/*rzxB^+^*: 8-fold mutant complemented with *rzxB;* Δ8-fold/*rzxB^+^phlA*^+^ : 8-fold mutant complemented with *rzxB* and *phlA*; Δ8-fold/*phlA^+^*: 8-fold mutant complemented with *phlA*; Δ8-fold/*prnC^+^*: 8-fold mutant complemented with *prnC*; Δ8-fold/*pltA^+^*: 8-fold mutant complemented with *pltA*; Δ8-fold/*ofaA^+^*: 8-fold mutant complemented with *ofaA*; Δ8-fold/*toxD^+^*: 8-fold mutant complemented with *toxD*; Δ8-fold/*pgnE^+^*: 8-fold mutant complemented with *pgnE*; Δ8-fold/*hcnB^+^*: 8-fold mutant complemented with *hcnB*. Picture is a representative of three replicates. The experiment was repeated three times. The radius of the inhibitory zone represents the mean ± standard deviation of three replicates. Different letters indicate significant differences (ANOVA, Tukey’s test, *P* < 0.05). (**B**) Mutation (via deletion or insertion) of the eight antibiotic loci in LK892 (Δ8-fold mutant). Numbers indicate locations in Pf-5 genome. (**C**) Impact of Pf-5 and its derivative lacking hydrogen cyanide on *D. pinodes*. Bacteria were cultured on KB agar which was separated from the fungal cultures on PMA. Fungal colony diameter at 14 days post-inoculation is presented as mean ± standard deviation of three replicates. The change of picric acid paper to blue or purple indicates hydrogen cyanide production. (**D**) Validation of the complemented strains of Pf-5. Restored production of DAPG, pyoluteorin and pyrrolnitrin was confirmed via HPLC analysis. Restored DAPG production was further tested via inhibition of *A. euteiches* on PDA plates. Restored production of toxoflavin and polyyne was tested via inhibition of *P. syringae* on KB plus FeCl_3_ plates and NA plus glycerol plates, respectively. Restored orfamide A production was tested via a swarming motility assay. The experiments were repeated at least two times with similar results.

To identify bacterial metabolites responsible for the growth inhibition of *D. pinodes*, *P. protegens* Pf-5 was used as a model because of its strong antagonistic activity (Fig. 5) and well-characterized antimicrobial biosynthetic pathways. The deletion of *gacA* abolished Pf-5-mediated inhibition of *D. pinodes* (Fig. 6A), indicating that inhibition depends on GacA-regulated antimicrobial compounds. Pf-5 is known to produce at least eight GacA-regulated antimicrobial metabolites (20, 30, 32, 33). To determine whether these compounds contribute to fungal inhibition, an eightfold deletion mutant lacking all corresponding biosynthetic genes (Δ8-fold mutant; strain LK892) was constructed and confirmed by whole-genome sequencing (Fig. 6B). The Δ8-fold mutant failed to inhibit *D. pinodes* (Fig. 6A), suggesting that at least one of the antimicrobial compounds is required for inhibition.

To identify the specific compounds involved, each deleted biosynthetic gene was individually complemented in the Δ8-fold mutant background, generating eight complemented strains that were evaluated for antagonistic activity against *D. pinodes*. Complementation of *rzxB* (rhizoxin biosynthesis) restored inhibition, generating the strain Δ8-fold/*rzxB*^+^ that clearly suppressed fungal growth (Fig. 6A). Similarly, complementation of *phlA* (DAPG biosynthesis) restored inhibition. Simultaneous complementation of both *rzxB* and *phlA* (Δ8-fold/*rzxB*^+^*phlA*^+^) also resulted in effective suppression of *D. pinodes*. Complementation of *prnC* (pyrrolnitrin biosynthesis) restored inhibitory activity although the resulting inhibition zone was smaller than those observed for Δ8-fold/*phlA*^+^ and Δ8-fold/*rzxB*^+^.

Among the Pf-5-derived antimicrobials, hydrogen cyanide (HCN) is a volatile compound. Restored HCN production of the complemented strain Δ8-fold/*hcnB*^+^ was confirmed via a colorimetric assay (Fig. 6C). The inhibition activity of Δ8-fold/*hcnB*^+^ was evaluated by co-culturing the bacterium with *D. pinodes* in a sealed plate assay. Results show that the colony size of *D. pinodes* was significantly decreased by co-culturing with the wild-type Pf-5 than the no-bacteria control (Fig. 6C), indicating Pf-5 produced volatile compound(s) to inhibit the fungus. The Δ*gacA* mutant and the Δ8-fold mutant had a less inhibition than the wild-type Pf-5. Importantly, strain Δ8-fold/*hcnB*^+^ showed a fungal inhibition activity which is similar to the wild-type Pf-5.

Furthermore, individual complementation of the biosynthesis genes *toxD*, *ofaA*, *pgnE,* and *pltA*, that are required for the production of toxoflavin, orfamide A, polyyne, and pyoluteorin, respectively, failed to restore inhibition against *D. pinodes* (Fig. 6A). Successful restoration of these antimicrobial compounds in the complemented strains was confirmed via phenotypic assays and HPLC analysis (Fig. 6D). For example, HPLC analysis showed that Δ8-fold/*phlA*^+^, Δ8-fold/*pltA*^+^, and Δ8-fold/*prnC*^+^ produced DAPG, pyoluteorin, and pyrrolnitrin, respectively.

Collectively, these results demonstrate that biosynthetic genes for rhizoxin, DAPG, pyrrolnitrin, and hydrogen cyanide are required for Pf-5-mediated inhibition of *D. pinodes*, indicating that these antimicrobial compounds contribute to pathogen suppression in cultures.

### Rhizoxin and DAPG contribute to the biocontrol of *P. protegens* Pf-5 against *D. pinodes*

To investigate the roles of antimicrobial compounds in Pf-5-mediated biocontrol of pea AB, biocontrol efficacy was evaluated using Pf-5 and its derivative strains. Compared with the wild-type Pf-5, both the Δ*gacA* and Δ8-fold mutants exhibited significantly reduced disease suppression, indicating that antimicrobial compounds regulated by GacA contribute to the biocontrol of pea AB.

**Fig 7:**
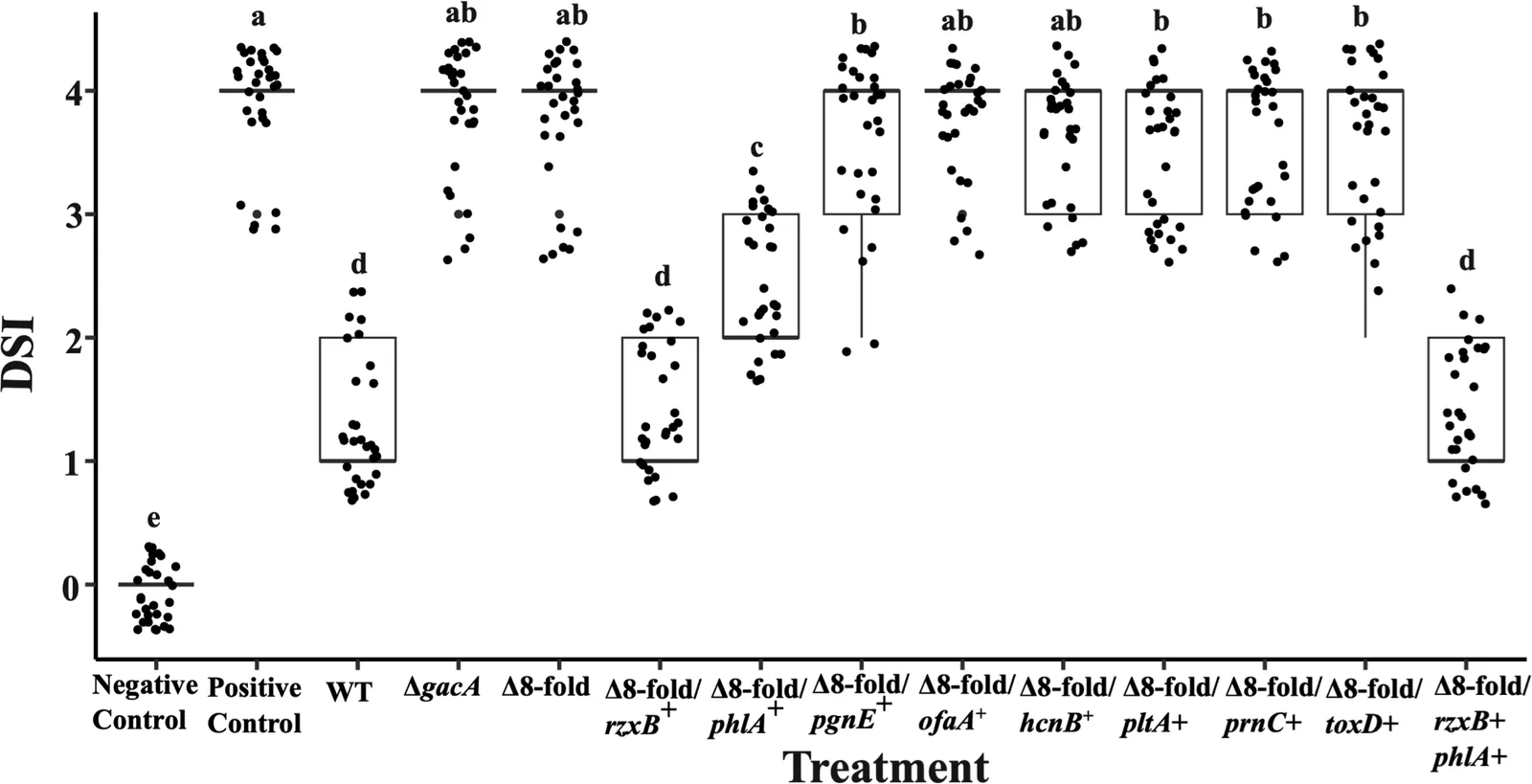
Biocontrol activity of *P. protegens* Pf-5 and its derivatives against *D. pinodes* under greenhouse conditions. Disease severity index (DSI) of each treatment was evaluated 7 days post fungal inoculation. The experiment was repeated independently 3 times, with 10 replicates per experiment. WT: wild type; Δ8-fold: Δ*prnC*Δ*rzxB*Δ*pltA*Δ*hcnB*Δ*ofaA*Δ*phlA*Δ*pgnE*Δ*toxC*; Δ8-fold/*rzxB^+^*: 8-fold mutant complemented with *rzxB;* Δ8-fold/*phlA^+^*: 8-fold mutant complemented with *phlA*; Δ8-fold/*pgnE^+^*: 8-fold mutant complemented with *pgnE*; Δ8-fold/*ofaA^+^*: 8-fold mutant complemented with *ofaA*; Δ8-fold/*hcnB^+^*: 8-fold mutant complemented with *hcnB*; Δ8-fold/*pltA^+^*: 8-fold mutant complemented with *pltA*; Δ8-fold/*prnC^+^*: 8-fold mutant complemented with *prnC*; Δ8-fold/*toxD^+^*: 8-fold mutant complemented with *toxD*; Δ8-fold/*rzxB^+^phlA* +: 8-fold mutant complemented with both *rzxB* and *phlA*. Data are presented as means ± standard deviation. Different letters indicate statistically significant differences among treatments (ANOVA, Kruskal-Wallis, *P* < 0.05).

Complementation of *rzxB* or *phlA* in the Δ8-fold mutant significantly enhanced biocontrol activity, as evidenced by reduced disease symptoms compared with plants treated with the Δ8-fold mutant alone (Fig. 7). Plants treated with the complemented strain Δ8-fold/*rzxB*^+^ showed no significant difference in DSI relative to those treated with wild-type Pf-5, indicating a full restoration of biocontrol activity. Complementation of *phlA* (Δ8-fold/*phlA*^+^) increased disease suppression although the DSI was higher than in plants treated with wild-type Pf-5, suggesting a partial restoration of biocontrol efficacy. Similarly, the double-complemented strain Δ8-fold/*rzxB*^+^*phlA*^+^ exhibited biocontrol activity comparable to that of the wild type.

Complementation of genes corresponding to the other six antimicrobial compounds in the Δ8-fold mutant did not restore biocontrol activity, indicating that these genes are not required for Pf-5-mediated control of pea AB.

Overall, these results demonstrate that *rzxB* and *phlA* are required for the biocontrol activity of Pf-5 against *D. pinodes*, suggesting that rhizoxin and DAPG contribute to the suppression of the pathogen *in planta*.

## DISCUSSION

In this study, beneficial bacteria belonging to multiple genera, including *Pseudomonas*, *Bacillus*, *Paenibacillus*, and *Peribacillus*, were identified as effective suppressors of pea AB in greenhouse pot assays. Using *P. protegens* Pf-5 and derivative strains deficient in, or producing, specific antimicrobial compounds, we demonstrated that rhizoxin and DAPG are required for Pf-5 to inhibit *D. pinodes in vitro* and to suppress the AB pathogen on pea leaves *in planta*. To our knowledge, this is the first report demonstrating that rhizoxin produced by beneficial bacteria contributes to the biocontrol of a foliar disease.

Rhizoxin is a macrocyclic polyketide originally identified as a virulence factor of *Rhizopus microsporus* (formerly *Rhizopus chinensis*), the causal agent of rice seedling blight (49). Subsequent work revealed that rhizoxin is produced by the endosymbiotic bacterium *Mycetohabitans rhizoxinica* (syn. *Burkholderia rhizoxinica*) rather than by the fungus itself (50). Rhizoxin exhibits potent antimitotic activity against a broad range of eukaryotes, including plants (49), fungi (51), and insects (41). Although rhizoxin has historically been studied as a virulence factor in pathogenic interactions, our results indicate that it can also function in beneficial plant-microbe interactions by suppressing fungal pathogens.

Consistent with our findings, *Pseudomonas* sp. Os17, a beneficial bacterium isolated from the rice rhizosphere, was reported to produce rhizoxin to control damping-off caused by *Pythium ultimum* and root rot caused by *Fusarium oxysporum* (52). Together, these studies suggest that microbial compounds traditionally regarded as virulence factors may also contribute to beneficial outcomes in plant-microbe interactions. Similarly, the bacterial type III secretion system, classically viewed as a virulence determinant mediating effector delivery by plant pathogens, has been shown to facilitate plant colonization by beneficial bacteria (53).

The observation that rhizoxin-producing strains, including wild-type Pf-5 and the complemented strain Δ8-fold/*rzxB*^+^, significantly reduced AB disease severity without causing visible phytotoxicity is particularly noteworthy. This finding suggests that Pf-5 produces rhizoxin on pea leaves at concentrations sufficient to inhibit the pathogen but below the threshold of plant toxicity. Alternatively, pea leaves may be relatively tolerant to rhizoxin produced by Pf-5. Supporting this possibility, a previous study reported that pea roots are less sensitive to Pf-5-derived rhizoxin than rice and cucumber roots (29).

In addition to rhizoxin, DAPG also appears to be produced by Pf-5 on pea leaves, as evidenced by the suppression of *D. pinodes* growth and reduced AB severity in plants treated with DAPG-producing Pf-5 derivatives (e.g., Δ8-fold/*phlA*^+^), but not with DAPG-deficient strains (Fig. 7). Although DAPG-producing bacteria are known to colonize leaf surfaces, the role of DAPG in the biocontrol of foliar diseases has been relatively understudied. Consistent with our results, DAPG was shown to be required for Pf-5-mediated suppression of leaf mold caused by *Botrytis cinerea* in cannabis (54).

DAPG is produced by many *Pseudomonas* species (55) and exhibits broad-spectrum antimicrobial activity against a wide range of plant pathogens, including oomycetes (21, 56–58), fungi (59, 60), bacteria (48), and nematodes (61). Research on DAPG has historically focused on rhizosphere processes and root disease management (62, 63). Our study, together with previous work (53), supports a broader role for DAPG in the biocontrol of foliar diseases. It is worth noting that DAPG also controls plant pathogens indirectly by triggering plant defense responses. For example, application of DAPG to soil or artificial plant growth media led to induced systemic resistance (ISR) in *Arabidopsis thaliana* against *Pseudomonas syringae* and *Botrytis cinerea* (64, 65). The current study does not exclude the possibility that DAPG produced by Pf-5 on pea leaves may activate plant ISR and contribute to the biocontrol of *D. pinodes*.

The observation that the DAPG-producing strain Δ8-fold/*phlA*^+^ exhibited lower biocontrol efficacy than wild-type Pf-5 and the rhizoxin-producing strain Δ8-fold/*rzxB*^+^ (Fig. 7) suggests that rhizoxin is more effective than DAPG in controlling pea AB. This difference may reflect a higher sensitivity of *D. pinodes* to rhizoxin than to DAPG or differential production levels of these compounds on pea leaves. Rhizoxin and DAPG act through distinct mechanisms: rhizoxin binds β-tubulin and disrupts cell division (66), whereas DAPG compromises membrane integrity and induces reactive oxygen species production (67). The combined use of two antimicrobial compounds with different modes of action likely enhances the robustness and consistency of disease suppression by Pf-5. Future studies should investigate whether rhizoxin and DAPG act additively or synergistically against *D. pinodes*.

Although the identified beneficial bacteria protected pea leaves from *D. pinodes* infection for up to 14 days, their biocontrol efficacy declined over time (Fig. 2). This decline was positively correlated with reduced bacterial populations on leaf surfaces, likely due to the harsh phyllosphere environment, which is nutrient-poor, UV-intensive, and characterized by rapid fluctuations in moisture and temperature (68, 69). Similar declines in population size and biocontrol efficacy have been reported for *Bacillus subtilis* QST 713 in the control of tomato early blight (70). Because foliar pathogens such as *D. pinodes* can infect host plants throughout the growing season, repeated application of biocontrol agents and integration with complementary disease management strategies that enhance bacterial persistence on leaves will likely be necessary for effective disease control (71).

Co-inoculation of beneficial bacteria did not enhance biocontrol efficacy against pea AB (Fig. 3). Similar outcomes have been reported in other foliar systems, where mixed bacterial inoculations often resulted in reduced disease suppression or diminished plant growth promotion compared with single-strain applications (72). Such reduced performance likely reflects incompatibility among strains due to competition for limited leaf nutrients and/or antagonistic interactions. These interactions can negatively affect bacterial growth and antibiotic biosynthesis, resulting in unchanged or even reduced disease control. In contrast, bacterial consortia frequently show additive or synergistic effects in root disease suppression (73–75), possibly due to greater nutrient availability and spatial heterogeneity in the rhizosphere that promote functional complementarity among strains (76). Together, these findings underscore fundamental ecological differences between leaf and root environments that strongly influence microbial interactions and biocontrol outcomes.

Beyond antibiotic production, beneficial bacteria can suppress plant pathogens through additional mechanisms, including competition for nutrients and space (77, 78), induction of plant defense responses (79), and promotion of plant growth (80, 81). *Peribacillus* sp. LK832 did not inhibit *D. pinodes in vitro* (Fig. 5) but significantly reduced AB severity *in planta* (Fig. 2A), suggesting that it suppresses disease through mechanisms other than direct antibiosis. Members of the genus *Peribacillus* are known to possess diverse traits relevant to agricultural applications (82). For example, *Peribacillus butanolivorans* KJ40 suppressed cucumber anthracnose by inducing systemic resistance (83), while *P. frigoritolerans* strains have been shown to promote plant growth in *Arabidopsis thaliana* and canola (84, 85). Further research is needed to elucidate the mechanisms employed by *Peribacillus* sp. LK832 and other beneficial bacteria identified in this study to control pea Ascochyta blight.

In summary, this study provides new insights into the biocontrol mechanisms of pea AB caused by *D. pinodes*. Using the beneficial bacterium *P. protegens* Pf-5 as a model, we show that *D. pinodes* can be inhibited by several bacteria-derived antimicrobial compounds including DAPG, rhizoxin, pyrrolnitrin, and hydrogen cyanide. More importantly, DAPG and rhizoxin play important roles in the biocontrol activity of Pf-5 *in planta*. This study also identified indigenous Montana bacterial strains with potential as biofungicides to control pea AB for local growers.

## Data Availability

The data obtained in this study are available upon request or can be accessed with individual gene accession numbers of the identified beneficial bacteria as shown in Fig. 2D.
